# Calibration, Compensation and Accuracy Analysis of Circular Grating Used in Single Gimbal Control Moment Gyroscope

**DOI:** 10.3390/s20051458

**Published:** 2020-03-06

**Authors:** Yue Yu, Lu Dai, Mao-Sheng Chen, Ling-Bo Kong, Chao-Qun Wang, Zhi-Peng Xue

**Affiliations:** 1 Changchun Institute of Optics, Fine Mechanics and Physics, Chinese Academy of Sciences, Changchun 130033, China; yuyuehfut@163.com (Y.Y.); chenms0911@aliyun.com (M.-S.C.); xuezhipengss1314@126.com (Z.-P.X.); 2 University of Chinese Academy of Sciences, Beijing 100049, China; 3 Chang Guang Satellite Technology Co., LTD, Changchun 130102, China; kongling_bo@163.com (L.-B.K.); hitwangchaoqun@163.com (C.-Q.W.)

**Keywords:** single gimbal control moment gyroscope, circular grating, error, eccentricity, accuracy compensation

## Abstract

The accuracy of the circular grating is the key point for control precision of the single gimbal control moment gyroscope servo system used in civilian micro-agile satellites. Instead of using the multi reading heads to eliminate eccentricity errors, an algorithm compensation method based on a calibration experiment using a single reading head was proposed to realize a low-cost and high accuracy angular position measurement. Moreover, the traditional hardware compensation method using double reading heads was also developed for comparison. Firstly, the single gimbal control moment gyroscope system of satellites was introduced. Then, the errors caused by the installation of the reading head were studied and the mathematic models of these errors were developed. In order to construct the compensation function, a calibration experiment using the autocollimator and 24-sided prism was performed. Comparison of angle error compensation using the algorithm and hardware method was presented, and results showed that the algorithm compensation method proposed by this paper achieved the same accuracy level as the hardware method. Finally, the proposed method was further verified through a control system simulation.

## 1. Introduction

The attitude control for unmanned systems [1] and an aerospace system are very important, for the precision of the attitude control has great influence on the accuracy and reliability of the system [2]. Single gimbal control moment gyroscope (SGCMG) is a critical system for the attitude control of a space system that can offer significant accuracy and efficiency control torque for the attitude adjustment and stability of the spacecraft. Its application in spacecraft attitude control has always been a research hot spot [3,4,5]. The output of the rotor used in SGCMG is a constant angular momentum, and the control accuracy mainly depends on the accuracy of the gimbal servo control system. Therefore, the accuracy of the output torque has a significant impact on the performance of attitude control. Generally, the influence of electromagnetic signal on the sensor accuracy of aircraft and spacecraft systems are very small [6]. In recent years, in order to improve the accuracy of servo control systems, many scholars have focused on servo control algorithms development including robust iterative learning control via adaptive sliding mode control [7], a variable structure controller and an adaptive feed forward controller [8] and a cascade extended state observer [9].

The circular grating is an angular position sensor of the SGCMG control system for measuring the angular position of the gimbal, and the angular velocity is further obtained by the differential processing of sensor signal. Generally, the circular grating with high-resolution and high-precision are used as a system sensor of the satellite to achieve high system accuracy. However, the errors caused by machining and installation [10,11,12] will have significant impact on the measurement accuracy of the circular grating. Improving the measurement accuracy of the circular grating is still an active research subject in various industrial fields. Dateng Zheng et al. [13] developed the 6 circular grating eccentricity errors model of an articulated arm coordinate measuring machine (AACMM). They also perform calibration and error compensation to improve the measurement accuracy. Ming Chu et al. [14] proposed a method for circular grating eccentric testing and error compensation for robot joint using double reading heads. Guanbin Gao et al. [15] studied the mounting eccentric error of the circular grating angle sensors and proposed a compensation method to compensate the error of the joints of an articulated arm coordinate measuring machine. For self-errors of the angle measuring sensor, such as sub-divisional error, Jiawei Yu et al. [16] established mathematical models for different types of sub-division errors of photoelectric angle encoders. They also proposed an algorithm compensation method based on the established models to improve the tracking precision of a telescope control system. Xianjun Wang [17] analyzed the cause of angle measuring error of grating for large telescopes and compensated the angle measuring error by resonant equation. Liandong Yu [18] proposed the harmonic method to compensate for the circular grating angle measurement error of the portable articulated coordinate measurement instrument machines caused by ambient temperature change. Li Xuan et al. [19] presented a spider-web-patterned scale grating to realize the eccentricity self-detection of the optical rotary encoder by a dual-head scanning unit.

However, the above compensated methods were developed for the ground system and the additional mass and power needed by these methods were not considered. The SGCMG system studied in this paper was used in the civilian micro-agile satellites. The mass of the whole satellite is less than 40 kg. Therefore, the requirements of the SGCMG system used in civilian micro-agile satellites are low mass, low power consumption, low cost and high precision. In general, the methods proposed by previous studies for compensating the eccentricity error of circular grating can be summarized as the hardware [20] and algorithm [21] compensation method. The hardware compensation method used multiple reading heads, which were symmetrical, mounted about the center of the circular grating to eliminate the eccentricity error [22,23,24]. The algorithm compensation method developed the compensation model, and the parameters of the model were obtained by the calibration experiments [25,26]. The method of using multi reading heads to eliminate eccentricity errors is easy to implement with high precision. However, for there are four SGCMG used in the satellite studied in this paper, and the compensation method should ensure accuracy requirements with relatively low power and mass. Moreover, few studies have focused on the effect of measurement accuracy on the performance of the servo control system, which is especially important for the SGCMG system development.

In general, the former research of scholars can be summarized as:

(a) Most researchers have focused on the circular grating’s accuracy of ground systems such as the articulated arm coordinate measuring machine (AACMM) and telescope control system.
(b) Many scholars studied the compensation methods and self-calibration [27,28] of circular grating by multi reading heads. However, few research studies concerning the algorithm compensation can be found in recent years, and the comparison of the algorithm and hardware method has not been the focus of research on compensation of the circular grating.
(c) A search of the literature revealed few studies that improved the performance of the servo control system that considered the accuracy of the circular grating sensors.

The contribution of this paper is to propose an error compensation method for the angle measurement of the SGCMG system used in satellite. Only one reading head was used and the angle measurement errors were compensated based on the calibration experiment to verify the accuracy of the algorithm method was almost the same as the hardware method. Moreover, a SGCMG servo system model for velocity control accuracy simulation was investigated to prove that improving the accuracy of circular grating can improve the accuracy of the servo control system. In the next section the design of a single gimbal control moment gyroscope system is described. In Section 3, the source of circular grating errors was studied, and the mathematic models of tilt and eccentricity error are also presented. Section 4 introduces the circular grating calibration experiment. Detailed results of the calibration experiment and accuracy analysis of the proposed method are given in Section 5, followed by conclusions in Section 6.

## 2. SGCMG System Modeling

### 2.1. Introduction of the SGCMG System

**Figure 1:**
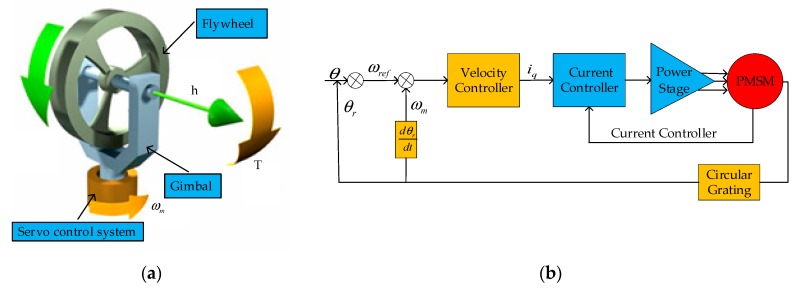
(**a**) System working principle diagram; (**b**) SGCMG gimbal servo control system structure diagram.

The SGCMG consists of constant-speed angular momentum flywheel, gimbal and servo control system, as shown in Figure 1a. The flywheel that is orthogonally mounted on the uniaxial gimbal generates the constant angular momentum by rotary motion. The gimbal generates a gyroscopic torque by changing the axis of the flywheel. The mathematic function of the output torque of the SGCMG is given as

(1) $${T=\omega }_{m}\times \;h,$$

where *T* is the output torque; *h* represents the flywheel angular momentum; *ω_m_* is the angular velocity of the gimbal. 

The angular momentum of the flywheel is maintained at a constant 5 Nms. Then the direction and value of the output torque depends on the angular velocity vector, which is the differential of the angle value measured by the circular grating sensor.

### 2.2. Control System

The servo control system of the SGCMG consists of one plant, one controller and two sensors. The plant is the system under control, which consists of a motor and mechanical structure. The mechanical structure is driven by a permanent magnet synchronous motor (PMSM) [29,30] that must follow the desired velocity profile. The current sensor and circular grating sensor are used to provide the feedback of the plant to controller.

As shown in Figure 1b, the servo controller consists of a current control loop and a velocity control loop. The servo system outputs a velocity reference signal *ω_ref_* according to the desired and actual angular position of the SGCMG system. The velocity controller compares the commanded velocity to actual velocity to increase or decrease the motor by generating the current command *i_q_* to current controller. 

For modeling of the SGCMG gimbal servo system, few assumptions were made as follows: (1) The PMSM iron core is unsaturated, and the eddy currents and hysteresis losses are negligible. (2) The stator windings are strictly three-phase symmetrically distributed, and the winding axes are spatially different from each other by 120° electrical angle. (3) There is no damped winding on permanent magnet rotor. (4) The induced electromotive force of the stator winding changes according to a strict sinusoidal law, ignoring the higher harmonic magnetic potential in the magnetic field. 

The equation of PMSM dynamic model can be expressed as:

(2) $$\frac{d\omega_{m}}{dt}=\frac{1}{J}(T_{e}-B\omega_{m}),$$

where *J* is the load rotating inertia, *B* is the viscous friction coefficient and *T_e_* is the electromagnetic torque, which can be given as:

(3) $$T_{e}=\frac{3}{2}P_{n}\psi_{f}i_{q},$$

in which *P_n_* is the number of magnetic poles on the rotor, *ψ_f_* is the permanent magnet flux linkage. The mathematical model of the calculation *i_q_* is as follows:

(4) $$i_{q}=\frac{u_{q}-\frac{d}{\mathrm{dt}}\psi_{q}-P_{n}\omega_{m}\psi_{f}}{R},$$

where $\psi_{q}=L_{q}i_{q}$ is the stator flux linkages, *L_q_* is the stator inductances, *u_q_* is the stator voltages, respectively, and R is the stator resistance. 

## 3. Eccentricity Error Modeling

**Figure 2:**
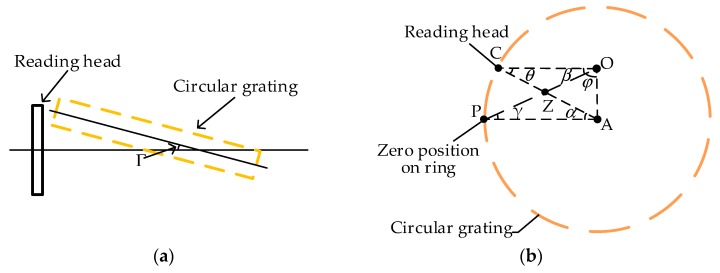
(**a**) Tilt error; (**b**) eccentricity error.

The circular grating error is the difference between the measured angle value of the reading head and the actual angle value. In general, the errors caused by the angle measurement of circular grating consists of two parts: self-errors and installation errors. The self-errors include the graduation accuracy and sub-divisional error [31], which are the periodic systematic errors that are sourced from the circular grating and the reading head. Generally, these errors can be decreased by using high-precision circular gratings and reading heads [32]. The installation errors mainly include the tilt and eccentricity error, which are caused by the installation of the circular grating and reading head. The grating tilt is caused by installation tilt and shaft sloshing, as shown in Figure 2a. The shaft sloshing is caused by the attitude control of the satellite and the micro-vibration of the flywheel rotating. The influence of tilt error on measurement accuracy is very small in comparison with eccentricity error. Therefore, tilt error is usually negligible. The mathematic model of tilt error is presented briefly in Appendix A.

The eccentricity error is caused by the non-coincidence between the geometric center of the circular grating and the rotational center of the measured shaft [33,34]. The value of the eccentricity error changes periodically with the rotation of the shaft. Because the eccentricity error is determined after the installation of the reading head and circular grating, the eccentricity error can be modeled by geometric method. Figure 2b shows the relationship between the actual rotation angle *β* of the circular grating and the measurement angle *α* of the reading head. A is the geometric center of the circular grating; O is the rotational center of measurement system; P is the zero angular position; C is the angle measuring position where the reading head is installed. Because the lines OP and AC intersect,

(5) β+θ=α+γ,

where *γ* and *θ* are the angle value of ∠OPA and ∠OCA, respectively.

According to the sine theorem, the relationship between side length and the angle of triangle ΔOAP and ΔOAC are given as

(6) $$\frac{\varepsilon }{\sin\gamma }=\frac{r}{\sin\varphi },$$

(7) $$\frac{\varepsilon }{\sin\theta }=\frac{r}{\sin(\varphi +\beta )},$$

where ***ε*** is the value of eccentricity (the distance between the geometric center and rotational center) and *r* is the radius of the circular grating; *φ* represents the direction of the eccentricity.

Then, *γ* and *θ* can be expressed as follows:

(8) $$\gamma =\arcsin(\frac{\varepsilon }{r}\sin\varphi ),$$

(9) $$\theta =\arcsin(\frac{\varepsilon }{r}\sin(\varphi +\beta )).$$

The grating measurement error of reading head is given as

(10) δ=α−β,

where *δ* is the measurement error of the circular grating.

By substituting Equations (5), (8) and (9) into (10), one can obtain,

(11) $$\delta =\arcsin(\frac{\varepsilon }{r}\sin(\varphi +\beta ))-\arcsin(\frac{\varepsilon }{r}\sin\varphi ).$$

If *r* is no less than 52 mm and the eccentricity ***ε*** is controlled within 0.01 mm, then

(12) $$\frac{\varepsilon }{r}\approx 0.$$

Based on the small-angle approximation theory, the simplification of the Equation (11) is given as

(13) $$\delta \;=\frac{\varepsilon }{r}\sin(\varphi +\beta )-\frac{\varepsilon }{r}\sin\varphi ,$$

where the sine value of the *δ* approaches 0, and the cosine value of *δ* approaches 1. Then sin(φ+β) is approximated as follows:

(14) $$\begin{array}{ll} \sin(\varphi +\beta ) & =\sin(\alpha -\delta +\varphi )\\ & =\sin(\alpha +\varphi )\cos(\delta )-\cos(\alpha +\varphi )\sin(\delta )\\ & \approx \sin(\alpha +\varphi ) \end{array}.$$

The Equation (13) can be reduced to

(15) $$\begin{array}{ll} \delta & =\frac{\varepsilon }{r}\sin(\alpha +\varphi )-\frac{\varepsilon }{r}\sin\varphi \\ & =\frac{\varepsilon }{r}\sin\alpha \cos\varphi +\frac{\varepsilon }{r}\sin\varphi (\cos\alpha -1)\\ & =M\sin\alpha +N(\cos\alpha -1) \end{array},$$

in which

(16) $$\left\{ \begin{array}{l} M=\frac{\varepsilon }{r}\cos\varphi \\ N=\frac{\varepsilon }{r}\sin\varphi \end{array}\right..$$

## 4. Circular Grating Error Compensation Method

### 4.1. Compensation Principle

**Figure 3:**
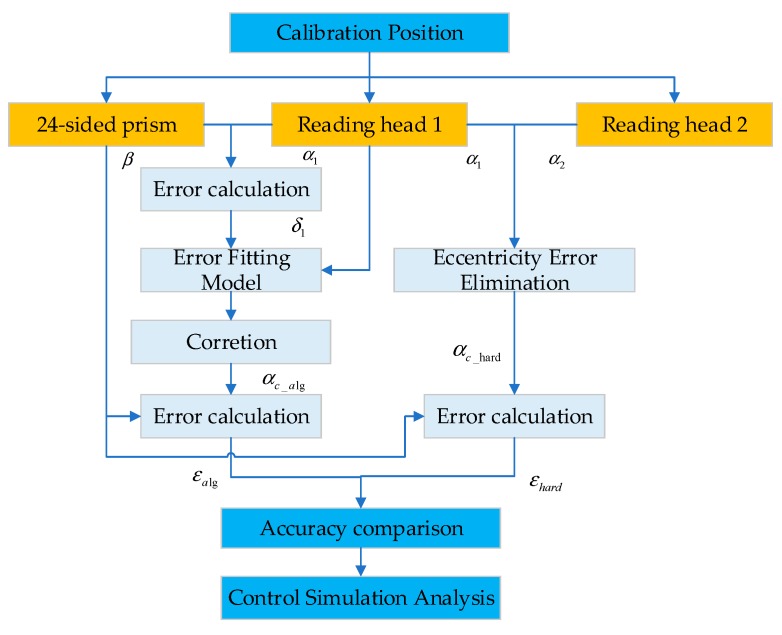
Circular grating error calibration process.

In order to achieve a relative high accuracy range by using the algorithm compensation method for the circular grating angle measurement in the SGCMG system of a satellite, a circular grating calibration and compensation system was proposed in this paper. Figure 3 shows the principle of measurement error calibration and compensation.

In order to prove a high precision angle measurement reference, the optical measurement method was also performed by using a 24-sided prism with the angle measurement accuracy 0.5″. The angular position is measured every 15° during the rotation of the circular grating. The reading head-prism calibration was performed to calculate the measurement error of reading head 1. Then, the algorithm compensation model was constructed by the fitting process. Finally, the constructed compensation model was used for the error correction of the circular grating.

(17) $$\alpha_{c\_a\mathrm{lg}}=\alpha_{1}-H(\alpha_{1}),$$

where *α_1_* is the measured value of reading head 1, *H(x)* is the algorithm compensation function and *α_c_alg_* is the angle value after algorithm compensation.

**Figure 4:**
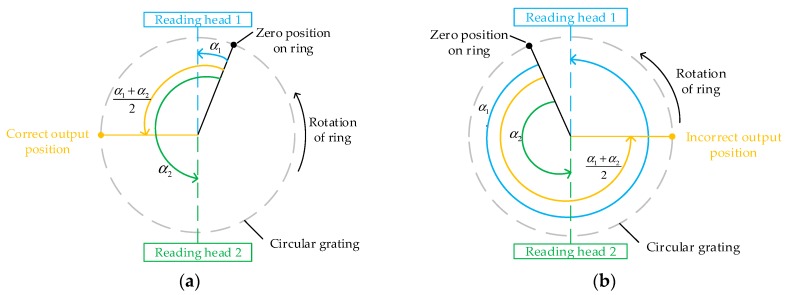
Double reading heads angle synthesis algorithm (**a**) *α_1_* ≤ *α_2_*, and (**b**) *α_1_* > *α_2_*.

Moreover, for the hardware compensation method, another reading head was installed on the circular grating, and the two reading heads were located symmetrically about the center of the circular grating. The principle of eccentricity error compensation using double or multiple reading heads was introduced in Reference [24]. As shown in Figure 4, the synthetic angle of the double reading heads can be given as

(18) $$\alpha_{c-hard}=\{\begin{array}{c} (\alpha_{1}+\alpha_{2})/2\\ (\alpha_{1}+\alpha_{2})/2+180^{{}^{\circ}} \end{array}\begin{array}{c} ,\alpha_{1}\leq \alpha_{2}\\ ,\alpha_{1}>\alpha_{2} \end{array},$$

where *α_1_* and *α_2_* are the angle value measured by reading head 1 and 2, respectively; *α_c-hard_* represents the compensated angle value using the hardware compensation method.

Finally, the accuracy of the algorithm and hardware compensation methods are calculated as

(19) $$\varepsilon_{i}=\alpha_{c\_i}-\beta ,$$

where *i* respects the algorithm (alg) or hardware (hard) compensation method; *ε_i_* represents the accuracy of method *i*; *α_c_i_* is the angle value after compensation using method *i*.

### 4.2. Calibration Experiment Setup

**Figure 5:**
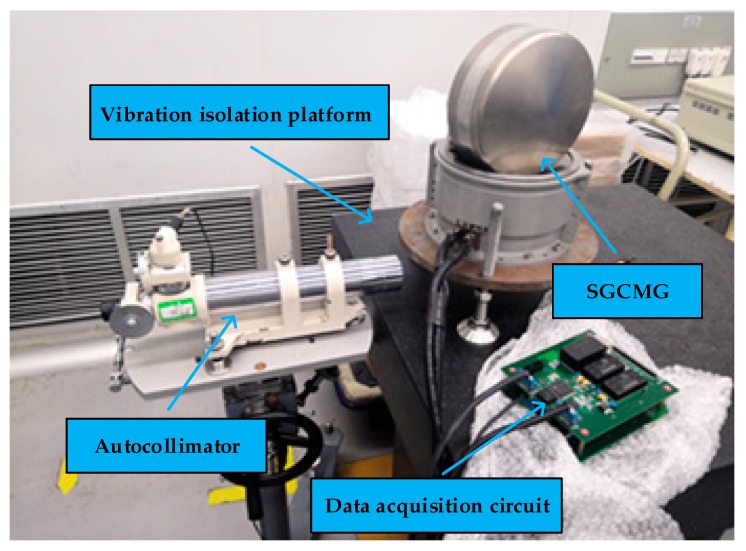
Calibration experiment.

**Figure 6:**
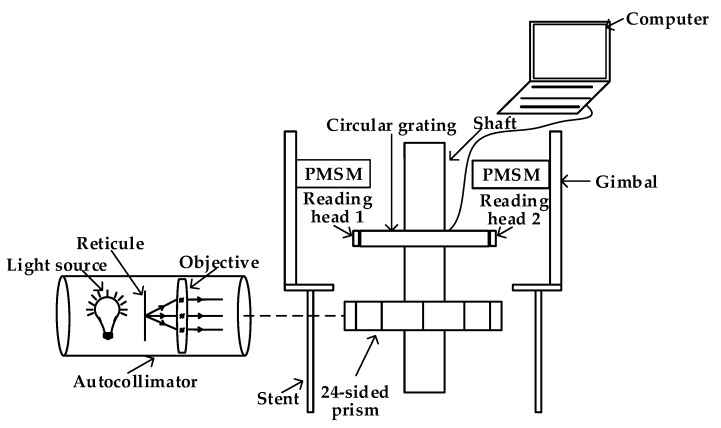
The schematic diagram of the calibration.

The experimental system includes an SGCMG system, autocollimator, 24-sided prism and vibration isolation platform, as shown in Figure 5. The schematic diagram of the experiment is shown in Figure 6. For isolating the vibration disturbance of the external environment, the SGCMG system was mounted on a vibration isolation platform. The autocollimator was used for generating parallel light and receiving the reflection optical signal of the 24-sided prism. The prism was mounted on the shaft of the SGCMG gimbal servo system. Note that it is critical to ensure that the optical axis of the light pipe is perpendicular to the prism’s surface. Reading head 1 and 2 were mounted on the circular grating symmetrically. The software for data recording and processing were also developed. The shaft system rotated 15° each time, and 24 angle positions were measured during the rotation of the system. The angle measuring values of the two reading heads and prism at each calibration position were recorded. Then both the algorithm and hardware compensation method were performed.

## 5. Results and Discussion

### 5.1. Measurement Error Compensation

#### 5.1.1. Test Data Processing

**Figure 7:**
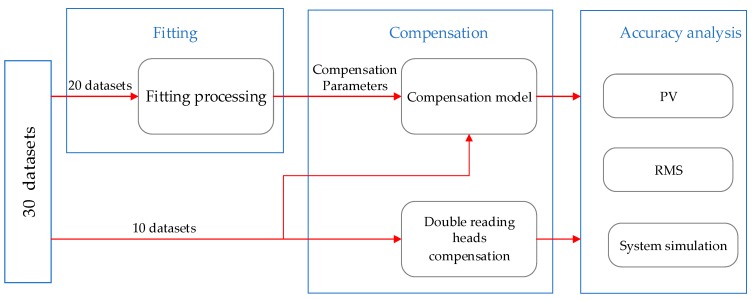
Data flow of experiment.

In order to obtain a higher accuracy compensation model, 30 sets of calibration experiments were performed separately, and the measuring values of the two reading heads and prism of the third calibration experiment are shown in Table 1. The main data flows of the data processing are shown in Figure 7. The results of the 30 experiments were divided into two datasets at first: 20 sets of experimental results for fitting (fitting dataset 67%) and 10 sets of experimental results for verification (testing dataset 33%). Then, the model of the algorithm compensation method was fitted using the 20 fitting datasets. Both the algorithm and hardware compensation (double reading heads) method were performed to correct the measurement errors of the 10 testing datasets. Furthermore, accuracy of the two methods were analyzed.

Based on Equation (15), the Fourier curve fitting is used to construct the compensation function of the algorithm method. The mathematical model of compensation function is given as

(20) $$\delta_{1}=a_{0}+a_{1}\sin(\omega \alpha_{1})+a_{2}\cos(\omega \alpha_{1}),$$

where the parameters of *a_0_*, *a_1_*, *a_2_* and *ω* of Equation (20) were determined by the fitting process through the measured values (α_1_, δ_1_) of the 20 fitting datasets. 

Table 2 shows the results of the fitting process. Statistical measure r-square was used to evaluate the prediction accuracy.

**Figure 8:**
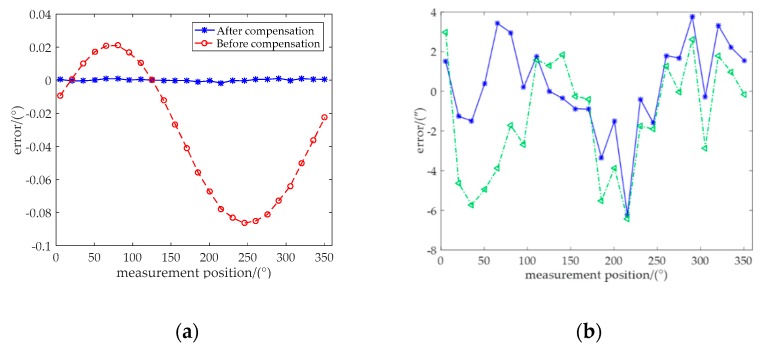
(**a**) Errors using one reading head before and after compensation. (**b**) Errors of algorithm and hardware compensation using one and double reading heads, respectively.

Then, the measurement errors of reading head 1 (δ_1_) of the 10 testing datasets were compensated based on the prediction values using the algorithm compensation method. Figure 8a shows the angle errors before and after the algorithm compensation of the first testing dataset. It is apparent that the algorithm was remarkably effective. Moreover, the hardware compensation method was also performed based on Equation (18). Both the compensation errors between the actual values (the angle values of prism) and predicted angle values of the algorithm and hardware compensation methods are shown in Figure 8b.

#### 5.1.2. Accuracy Analysis

In order to further identify the accuracy of the proposed method, both the peak-to-valley (PV) and Root Mean Square (RMS) errors of the angle errors before and after compensated are presented in Table 3. The system accuracy of circular grating used in this study, including the graduation accuracy and sub-divisional error, is 5.49″. The PV error of algorithm and hardware compensation method are 6.23″ and 6.43″, respectively. Therefore, a good agreement can be observed between the system accuracy of circular grating and the two compensation methods (13% and 17%). The errors caused by the installation of the circular grating were reduced significantly by the compensation methods. Moreover, the accuracy of the algorithm method was almost the same as with the hardware method. 

**Figure 9:**
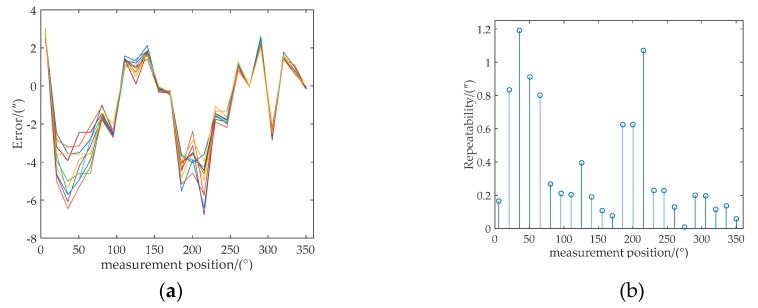
(**a**) The 10 testing datasets of compensation errors. (**b**) The repeatability at 24 angle measurement positions of compensation results.

The repeatability of the algorithm compensation method was also evaluated. The compensation errors of the 10 testing datasets using the algorithm compensation method are shown in Figure 9a. Repeatability [35] is the closeness between the results of successive measurements of the same measure carried out under the same conditions. Therefore, the repeatability *S_j_* of the circular grating compensation results at each measurement position is expressed as

(21) $$S_{j}=\sqrt{\frac{\sum_{i=1}^{n}{\left(\varepsilon_{\mathrm{ij}}-\overset{-}{\varepsilon_{j}}\right)}^{2}}{n-1}},$$

where *i* and *j* represent the number of the 10 testing datasets and 24 angle measurement positions, respectively; *ε_ij_* is the measurement error after algorithm compensation of the *j^th^* angle measurement position in *i^th^* testing datasets; ${\bar{\varepsilon }}_{j}$ is the average value of the measurement errors after compensation at each measurement position; *n* = 10 is the number of testing datasets.

The repeatability results of the 10 testing datasets at 24 angle measurement positions are shown in Figure 9b. In order to further analysis the accuracy of the algorithm compensation method, both the PV and RMS errors of the maximum of 10 testing datasets and repeatability are presented in Table 4. The maximum PV value of the 10 testing datasets is 6.78″. The maximum RMS value of the 10 testing datasets is 3.17″. Therefore, a good agreement can be observed between the repeatability experiment of the 10 testing datasets in Figure 9a and the algorithm compensation method in Figure 8. The maximum value of the repeatability of the compensation results using the algorithm method is 1.19″. The RMS value of the repeatability of the compensation results using the algorithm method is 0.50″.

### 5.2. SGCMG Simulation Results

**Figure 10:**
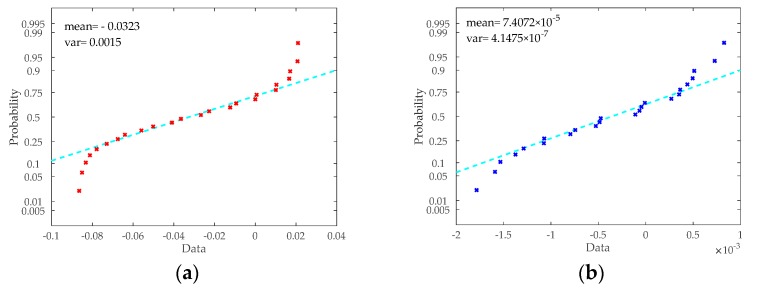
(**a**) Probability plot for normal distribution before compensation. (**b**) Probability plot for normal distribution after compensation.

In order to further verify the effect of the compensated measurement results on the control accuracy of the SGCMG system, the system simulation according to the model of control system introduced in Section 2 was also performed, and the parameters used in the simulation are shown in Table 5. Simulation 1 and 2 were performed considering the angular measurement error before and after compensation, respectively. The angle inputs of the control system were the sum of the angle outputs of the motor and the errors calculated by the error model at every simulation step. The probability distributions of the error before and after compensation are shown in Figure 10. It is apparent that both errors before and after compensation were approximately normal distribution. Then, random errors were generated from the normally distribution with the same mean and variance at every simulation step.

**Figure 11:**
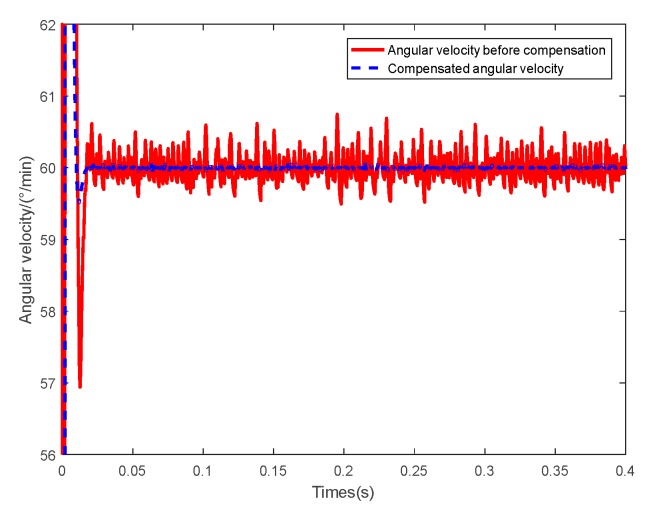
Angular velocity of motor.

The same control parameters were used in the two simulations, and the desired angular velocity of motor was 60 °/min. Figure 11 shows the angular velocity of the motors of the two simulations. The two simulations of accuracy of the control system are summarized in Table 6. The velocity of the motors of the two simulations were in the rang 59.50–60.75 °/min and 59.94–60.07 °/min respectively. The Velocity Root Mean Square Error (RMSE) was 0.0283 °/min and 0.0001 °/min, respectively. It is apparent that the control performance and accuracy was improved greatly by the compensation of the angle measurement error. 

## 6. Conclusions

In order to improve control precision of the SGCMG, a general and systematic methodology is presented to compensate the measurement error of circular grating. A calibration experiment was proposed to measure the error of the circular grating. The interactions among the measurement error, compensation accuracy and control accuracy were investigated. Both the algorithm and hardware compensation method were performed, and a comparison and appraisal were made for both methods. In general, therefore, it seems that the proposed method was effective, offering compensation for the measurement error of the circular grating with only one reading head. The results of this study indicate that the error calibration and compensation have achieved accuracy solutions for measuring and predicting the measurement error of circular grating. Based on the results of this study, the following main conclusion can be drawn:

(1) Eccentricity error is the main source of measurement error of circular grating.
(2) The key step in the proposed method is that the error calibration process includes calibration and fitting of the measurement error to provide accurate fitting compensation models to predict measurement errors.
(3) The accuracy of the algorithm method was almost same with the hardware method in this study. We also conducted fitting processing with a two-term Fourier compensation model, and the accuracy of the measurement was not obviously improved.

Generally speaking, we observe that the algorithm compensation method proposed in this paper effectively offers good potential to be applied to the angle measurement of circular grating used in the space system in order to meet the requirements of lower-mass and high accuracy.

## Figures and Tables

**Table 1:** Results of the circular grating angle measurement error (°).

Parameters	Value	Value	Value	Value	Value	Value	Value	Value
β	360	15	30	45	60	75	90	105
α_1_	359.9906	15.0024	30.0106	45.0181	60.0213	75.0215	90.0173	105.0111
α_2_	184.7492	199.73796	214.7281	229.7227	244.7199	259.7202	274.7228	289.7298
β	120	135	150	165	180	195	210	225
α_1_	120	134.9879	149.9737	164.959	179.9439	194.933	209.923	224.9187
α_2_	304.7384	319.751	334.7654	349.7793	4.7924	19.8052	34.8138	49.8216
β	240	255	270	285	300	315	330	345
α_1_	239.9138	254.9148	269.9197	284.9261	299.9368	314.9492	329.9633	344.977
α_2_	64.8247	79.8251	94.8217	109.8127	124.8029	139.7901	154.7771	169.7628

**Table 2:** Fitting results.

a_0_	a_1_	a_2_	ω	R-Square
−0.03201	−0.01573	−0.05181	1.858	99.97%

**Table 3:** Accuracy comparison.

Parameters	*α_1_*	*α_2_*	Algorithm Compensation	Hardware Compensation
PV	311.18″	312.12″	6.23″	6.43″
RMS	180.00″	180.36″	3.13″	2.29″

**Table 4:** Accuracy analysis.

Parameters	The 10 Testing Datasets	Repeatability
PV	6.78″	1.19″
RMS	3.17″	0.50″

**Table 5:** Parameters of PMSM.

Parameter	*P_n_*	*L_d_*	*L_q_*	*R*	*ψ_f_*	*B*	*J*
Value	4	1.5 mH	1.5 mH	0.011 Ω	0.077 Wb	0	0.0008 kg.m^2^

**Table 6:** Angular velocity tracking accuracy (°/min).

Simulation Num	Error Type	Velocity Range	RMSE
1	Before compensation	59.50–60.75	0.0283
2	After compensation	59.94–60.07	0.0001

## Appendix A. The Mathematic Model of Tilt Error

The maximum value of the moire fringes deflection during circular grating rotated can be expressed as

(A1) Δl=±rsinΓ,

where Γ is the angle of inclination of the circular grating relative to the plane of the reading head, and r is the radius of the circular grating.

The number of moire fringes deflection is given as

(A2) $$\Delta N=\frac{\pm r\sin\Gamma }{l},$$

in which l is the width of moire fringes.

(A3) $$l=\frac{g}{\Phi },$$

where g is grating pitch, and Φ is the grating line angle.

In the case of a circular grating rotated one of pitch g, the moire fringes will deflect one of stripe width l. Therefore, the amount of rotation *s* of the circular grating can be calculated by the number N of moire fringes deflection. The *s* can be expressed as

(A4) s=N⋅g=N⋅lΦ.

The angle of circular grating rotated is given as

(A5) $$\alpha =\frac{s}{r}=\frac{N\cdot l\Phi }{r}.$$

The mathematic model of tilt error can be obtained by substituting Equation (A2) into Equation (A5), which is given as

(A6) $$\Delta_{t}=\frac{\pm r\sin\Gamma }{l}\cdot \frac{l\Phi }{r}=\pm \Phi \sin\Gamma .$$
